# Evanston Letter

**Published:** 1869-06-15

**Authors:** J. H. Etheridge

**Affiliations:** Evanston, Ill.


Editor Chicago Medical Journal:

The following account of a case of aconite poisoning may not be uninteresting to your readers. The patient was a young man—twenty years old—had pneumonia. I was called to see him in great haste at 6:30 a. m., April 10th, and found that his attendants, contrary to directions, had given him medicine every (2) hours of the night, so that he got about eight drops of the tincture of aconite root each dose! Found him vomiting terribly at intervals of four or five minutes. Pulse fifty-six—was 114 the night previous; pupil dilated and jagged: surface cold; extremities nearly insensible; calling for something to stop his vomiting.

The nausea and vomiting came on suddenly; felt comfortable till it began; I gave him a hypodermic injection on the forearm of atropiæ sulph, gr. 1-40; and spirits frum., and aq. amm. internally. He vomited up a little greenish viscid fluid once, after administering the above. In ten minutes after the injection his vomiting and nausea all ceased; in twenty minutes more his pulse came up to 100, and in half an hour subsequently he was sleeping quietly, and continued sleeping for two hours. No further effects followed, except sore abdominal muscles. After giving the injection, and while I was preparing the whisky and amm. he said he felt the influence of the former and that it was quieting his stomach.

If I have another case of aconite poisoning I shall use atropia.

Respectfully yours, etc.,

J. H. ETHERIDGE, Evanston, Ill.

May 14th, 1869.

